# Incorporation of Chitosan in Polyurethanes Based on Modified Castor Oil for Cardiovascular Applications

**DOI:** 10.3390/polym15183733

**Published:** 2023-09-11

**Authors:** Maria Morales-González, Kelly Navas-Gómez, Luis E. Diaz, José A. Gómez-Tejedor, Manuel F. Valero

**Affiliations:** 1 Energy, Materials and Environmental Group (GEMA), Faculty of Engineering, Universidad de La Sabana, Chía 140013, Colombia; 2 Bioprospecting Research Group (GIBP), Faculty of Engineering, Universidad de La Sabana, Chía 140013, Colombia; 3 Centre for Biomaterials and Tissue Engineering, Universitat Politècnica de València, 46022 Valencia, Spain; 4 Biomedical Research Networking Centre in Bioengineering, Biomaterials and Nanomedicine (CIBER-BBN), 46022 Valencia, Spain

**Keywords:** polyurethane, castor oil, transesterification, chitosan, cardiovascular, hemocompatibility

## Abstract

The increased demand for vascular grafts for the treatment of cardiovascular diseases has led to the search for novel biomaterials that can achieve the properties of the tissue. According to this, the investigation of polyurethanes has been a promising approach to overcome the present limitations. However, some biological properties remain to be overcome, such as thrombogenicity and hemocompatibility, among others. This paper aims to synthesize polyurethanes based on castor oil and castor oil transesterified with triethanolamine (TEA) and pentaerythritol (PE) and with the incorporation of 1% chitosan. Analysis of the wettability, enzymatic degradation, mechanical properties (tensile strength and elongation at break), and thermal stability was performed. Along with the evaluation of the cytotoxicity against mouse fibroblast (L929) and human dermal fibroblast (HDFa) cells, the hemolysis rate and platelet adhesion were determined. The castor-oil-based polyurethanes with and without 1% chitosan posed hydrophobic surfaces and water absorptions of less than 2% and enzymatic degradation below 0.5%. Also, they were thermally stable until 300 °C, with tensile strength like cardiovascular tissues. The synthesized castor oil/chitosan polyurethanes are non-cytotoxic (cell viabilities above 80%) to L929 and HDFa cells and non-thrombogenic and non-hemolytic (less than 2%); therefore, they are suitable for cardiovascular applications.

## 1. Introduction

Cardiovascular diseases (CVDs) continue being the leading cause of death globally, with an estimated of 17.9 million deaths in 2019 [1]. Some of them are related to blockage of the arteries that demands vascular grafts [2]. To generate vascular grafts, biocompatible and blood-contacting materials are required to prevent post-surgical failure [3]. Due to this, research on different polymers has increased to achieve the requirements of vascular grafts.

Polyurethanes are one of the most synthetic polymers used as s biomaterial due to their controllable properties, such as mechanical and flexibility properties [4]. Along with their biological performance in terms of high biocompatibility, it makes them suitable for different biomedical applications like cardiovascular devices, bone and cartilage repair, catheters, and heath valves [5]. However, until now, many of the polyols used for its synthesis are from nonrenewable petrochemical resources, and considering sustainability and environmental friendliness, there is a necessity to find and develop environmentally friendly bio-based PU [4].

One of the interesting ways to apply more environmentally friendly polyols is the use of vegetable oils [6]. Castor oil has been investigated to generate polyurethane due to its chemical structure that provides hydroxyl groups for reaction with isocyanates [5], and generated PU has shown high thermal stability and desirable mechanical properties [7].

The Energy, Materials, and Environment Group and the Bioprospecting Research Group of the Universidad de La Sabana have been focused on the study of castor oil as a polyol for the synthesis of different polyurethanes that can be used in different applications. Different studies have demonstrated the high biocompatibility of the castor oil bio-based PU with fibroblasts and endothelial cells [8,9,10]. Also, in vivo studies performed in the groups with the grafting of the PU in the subcutaneous tissue of Wistar rats showed that there were no chronic lesions in the skin, indicating the safety of the polyurethanes from castor oil in contact with skin tissue [11].

Although satisfactory results have been obtained with castor oil in the biomedical field, some issues are still related to the properties of the PU, specifically using unmodified castor oil. Although castor oil contains hydroxyl groups in its structure, these are low in functionality, as they are secondary hydroxyl groups that have lower reactivity [12]. Therefore, modifications in castor oil could improve the functionality of the polyol and thus the properties of the synthesized PU. Modifications in castor oil can be carried out by transesterification to increase the functionality of ricinoleic acid triglycerides through the introduction of hydroxylic groups, which could improve the mechanical properties of the PUs [13].

Similarly, even though PU presents high biocompatibility, different issues regarding hemocompatibility have been found. Due to the hydrophilic characteristics of PU, its exhibit thrombogenicity in contact with blood, activating the coagulation cascade, platelet activation, and aggregation on the surface [14]. For example, Nguyen et al. made virgin-coconut-oil-loaded electrospun polyurethane/polycaprolactone membranes and evaluated the hemocompatibility, but the results showed activated platelets on the surface of PU/PCL composites and VCO6/PU/PCL samples; however, they said that the presence of a higher concentration of VCO prevented platelet activation and adherence to a target surface [15]. Thus, the inclusion of different compounds that can increase the hemocompatibility are required. Chitosan is a positively charged polysaccharide that has shown biocompatibility, biodegradability, and antimicrobial activity [16]. Specially, it promotes interaction with negatively charged groups that allow modification of the PU surfaces [17]. According to this, Drozd et al. synthetized layer-by-layer PU coating with chitosan and heparin and evaluated the clotting time of human blood and the platelet adhesion, finding that the activation of the surface of PU and the application of the layers of chitosan and heparin resulted in the appearance of anticoagulant potential of PUs [16].

In this paper, castor oil and transesterified castor oil were used as polyol for the synthesis of polyurethane, with the inclusion of chitosan in the polyurethane matrix in order to enhance the biocompatibility and hemocompatibility of the PUs.

## 2. Materials and Methods

### 2.1. Materials

Castor oil (CO) (*Ricinus communis*) was purchased from Químicos Campota and Co., Ltd. (Bogotá, Colombia). Isophorone diisocyanate (IPDI), low-molecular-weight chitosan (Ch, with a percentage of deacetylation between 75–85%), and porcine liver esterase (18 units mg^−1^) were acquired from Sigma-Aldrich Chemical Co. (St. Louis, MO, USA). Pentaerythritol was from Merck KGaA (Darmstadt, Germany). Triethanolamine (TEA) was obtained from BASF (Bogotá, Colombia). Phosphate-buffered saline (PBS: Dulbecco’s phosphate-buffered saline), 3-(4,5-dimethyl-2-thiazolyl)-2,5-diphenyl-2H-tetrazolium bromide (MTT), 2.5% trypsin (10×), and Dulbecco’s modified Eagle medium (DMEM, 1×) were obtained from Gibco/Invitrogen (Paisley, UK). Fetal bovine serum (FBS) was from Eurobio (Les Ulis, France). Triton^®^ X-100 was obtained from Thermo Scientific (Waltham, MA, USA). CytoTox 96^®^ Non-Radioactive Cytotoxicity Assay kit was provided by Promega (Woods Hollow Road, Madison, WI, USA). L-929 mouse subcutaneous connective tissue fibroblasts cells (ATCC^®^ CCL-1) and Adult human dermal fibroblast HDFa (ATCC^®^ PCS-201-012™) were also used.

### 2.2. Transesterification of Castor Oil

Two transesterifications of the castor oil were performed in a 500 mL reactor equipped with mechanical agitation, a thermometer, and a reflux condenser, with triethanolamine (T) and with pentaerythritol (P) [13]. For the transesterification with triethanolamine, 1.3% mol of triethanolamine per mole of CO was used, and the mixture was heated to 150 °C. Lead oxide (PbO) was added as a catalyzer at a ratio of 0.05% mass with respect to the mass of CO. The polyol obtained was labelled polyol (T1).

Transesterification with pentaerythritol was performed similarly, using 1.3% mol of pentaerythritol per mole of CO at 210 °C, and the obtained polyol was labelled (P1). The hydroxyl value of the polyols was obtained using the ASTM D-1957-86 guideline.

### 2.3. Synthesis of Polyurethanes

The polyurethanes were synthesized using the pre-polymer method in a reactor equipped with an agitator and temperature sensor. The required amount of each polyol (castor oil without modification (CO), T1, and P1) was brought to 60 °C at reduced pressure to eliminate traces of humidity. Then, the polyol was mixed with IPDI using a 1:1 NCO/OH ratio and maintained at 300 rpm for 5 min, followed by the addition of Ch (1% *w*/*w* of oil polyol weight) at 300 rpm for an additional 5 min. The PU’s formation was completed by pouring the prepolymer into a steel mold. Curing was performed at 110 °C for 12 h. PUs are identified with the following nomenclature: PnChx, where Pn represents the polyol used (CO, T1, and P1), and x represents the percentage of Ch.

Fourier-transform infrared spectroscopy (FTIR) were performed according to Uscátegui et al., 2019 [10].

### 2.4. Mechanical Tests

A universal testing machine EZ-LX (Shimadzu, Kyoto, Japan) was used to determine tensile strength and elongation at break of the PUs (following the ASTM D638-10 standard). A load cell of 5 kN with a crosshead speed of 25 mm min^−1^ was used. Three samples of 40 mm × 6 mm × 3 mm (length × width × thickness) were tested.

### 2.5. Thermal Analysis

The thermogravimetric analyses were according to Uscátegui et al., 2019 [11].

### 2.6. Hydrophilic Character

The sessile drop method was used for the contact angel measurement and was carried out following the procedures of Uscátegui et al., 2019 [11].

The water absorption was determined in distilled water at 8 days according to the procedure of Uscátegui et al., 2019 [10]. The rate of water absorption at equilibrium was calculated by comparing the mass of the sample (*w_t_*) with the initial mass (*w_i_*) of the sample using Equation (1):

(1) $$Water\;absoption\;rate\;\left(\%\right)=\frac{\left(w_{t}-w_{i}\right)}{w_{i}}\times 100\%$$

### 2.7. Enzymatic Degradation

The PUs were dried in a vacuum chamber at 37 °C for 24 h. Samples of 5 mm × 5 mm × 3 mm were placed in the enzymatic degradation media (porcine liver esterase enzymatic medium (10 units/mg solid)) and incubated at 37 °C for 21 days [18]. After this time, the samples were washed with distilled water, dried in a vacuum chamber, and weighed according to ASTM F1635-11. Six measurements of each material were performed. The degradation rate was calculated by comparing the dry weight (*w_t_*) of the sample after degradation with the initial dry weight (*w_i_*) of the sample using Equation (2):

(2) $$Degradation\;rate\;\left(\%\right)=\frac{\left(w_{t}-w_{i}\right)}{w_{i}}\times 100\%$$

### 2.8. Cell Culture

L929 and HDFa were cultured in DMEM supplemented with 10% FBS and 1% penicillin-streptomycin in T-75 cell culture flasks. They were grown at 37 °C and 5% CO_2_. The cell culture medium was changed every 48 h [19]. At 100% confluence, the cells were trypsinized (trypsin-EDTA) for viability analysis.

### 2.9. In Vitro Cell Viability Assay

The effect of PUs on L929 viability was evaluated by the LDH with a CytoTox 96^®^ Non-Radioactive Cytotoxicity Assay kit as per the manufacturer instructions.

For the effect of PUs on HDFa, viability was evaluated by the MTT method following the procedures of Uscateguí et al., 2019 [11]. As a positive control, polypropylene biomaterial (PP) was used, and latex and 10% DMSO were used as a negative control. Cell viability was determined according to Equation (3):

(3) $$Cell\;viability\;\left(\%\right)=\frac{{Abs}_{sample}}{{Abs}_{control}}\times 100\%$$

### 2.10. Hemolysis Assay

The hemolytic activity of all materials was evaluated with a hemolysis assay (ASTM F750-00) following the procedure of Raut et al., 2019 [20]. The absorbance of all supernatants was measured at 545 nm (*Abs_sample_*). For positive control, 0.5% Triton-X was used, while PBS was used as negative control. The hemolysis rate was computed as follows:

(4) $$Hemolysis\;rate\;\left(\%\right)=\frac{\left({Abs}_{sample}-{Abs}_{negative}\right)}{\left({Abs}_{positive}-{Abs}_{negative}\right)}\times 100\%$$

### 2.11. Platelet Adhesion

PUs were placed in 24-well plates, and 1 mL of platelet-rich plasma (PrP) was added to each well, followed by incubation at 37 °C for 1 h [20]. PrP was aspirated, and samples were washed twice with PBS to eliminate the non-adhered platelets on the surface of the material. The effect of PUs on platelet adhesion was evaluated by the LDH released by the adhered platelets with a CytoTox 96^®^ Non-Radioactive Cytotoxicity Assay kit as per the manufacturer instructions. The 100% control for the platelet’s adhesion was seeded on glass and subjected to the same process as the materials.

### 2.12. Statistical Analysis

The results are expressed as mean values ± standard deviation (SD) and analyzed by the one-way analysis of variance (ANOVA) to determine the statistical significance (*p* < 0.05). The Student’s *t*-test was used for the comparison between samples with the software SPSS Statistics Software v.23 (IBM, Armonk, NY, USA).

## 3. Results

**Figure 1:**
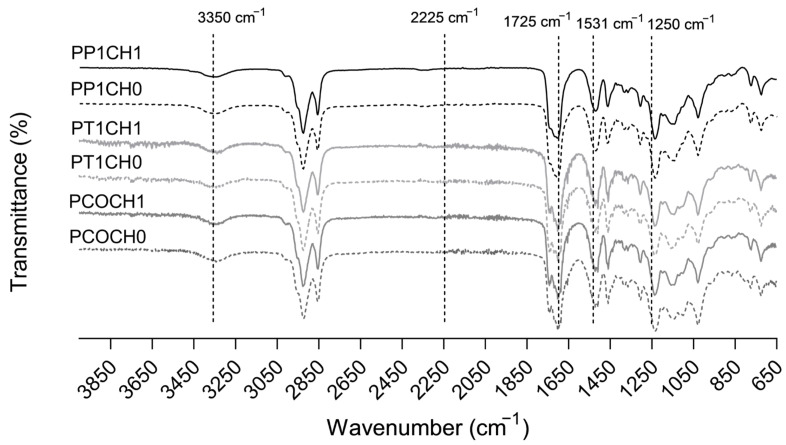
FTIR spectra of PUs synthetized with castor oil (CO), castor oil transesterified with TEA (T1), and castor oil transesterified with PE (P1) and with chitosan.

The FTIR spectra of synthesized PUs are shown in Figure 1. Bands at 3350 cm^−1^ (N-H stretching), 1725 cm^−1^ (C=O stretching), and 1531 cm^−1^ (N-H flexion) confirmed the obtention of polyurethanes [21]. Moreover, the absence of the band at 2225 cm^−1^ assigned to the −N=C=O bond of the isocyanate group suggests non-unreacted isocyanate and thus the complete reaction of IPDI [22]. Around 1250 cm^−1^, the C–N bond stretching is evidenced, and near 1140 cm^−1^, the stretching vibrations of the C–O bond are seen [22,23]. The values of the hydroxyl index for CO were 160, 178, and 191 mg KOH per g of castor oil sample, respectively.

**Figure 2:**
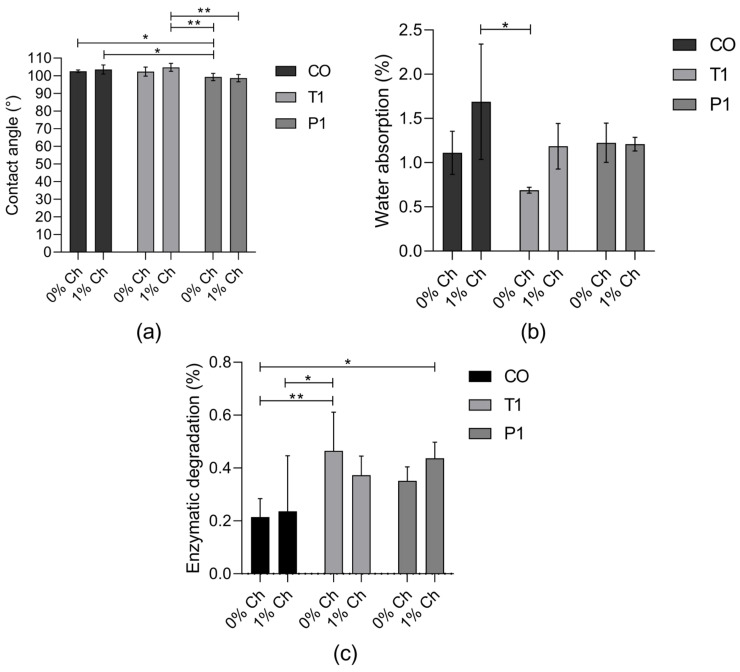
(**a**) Contact angle measurements (mean ± SD (*n* = 10)); (**b**) water absorption rate (%) over 192 h (mean ± SD (*n* = 3)); (**c**) enzymatic degradation rate (%) of PUs in with esterase for 21 days (mean ± SD (*n* = 6)). Bars with * indicates significant differences (*p* < 0.05); ** indicates significant differences (*p* < 0.01).

The surface wettability of PUs was analyzed with the measurements of contact angle values, and according to Figure 2a, a decrease in the contact angle of the PUs with 1% of chitosan was observed regarding the modification made in the castor oil. A surface can be considered hydrophobic if the contact angle with water is greater than 90° [16], and for the synthesized PUs, all surfaces were hydrophobic, with contact angles greater than 95° and lower than 105° (Figure 2a).

Contact angles less than 90° suggest that wetting of the surface is favorable [24], so the water absorption in the polyurethane will be greater. For the synthesized PUs (Figure 2b), the water absorption was between 0.68% and 1.68%. They reached the maximum water absorption in the first 5 h and remained stable during the 192 h. Related to this, the enzymatic degradation of PUs was lower than 0.47% after 21 days of incubation (Figure 2c).

**Figure 3:**
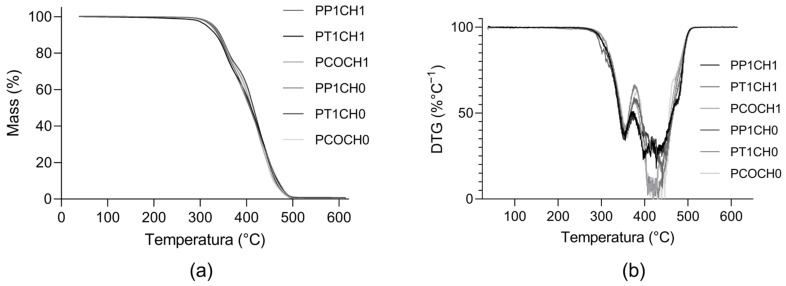
Thermograms of synthesized PUs. (**a**) Thermogravimetric (TG) curve of PUs and (**b**) derivative of the thermogravimetric (DTG) curve of PUs.

All the PUs presented similar thermal behavior: under 300 °C, the PUs presented thermal stability without mass loss and underwent two degradation steps (Figure 3). According to TG and DTG thermograms, the main weight loss occurred between 400 °C and 480 °C.

**Figure 4:**
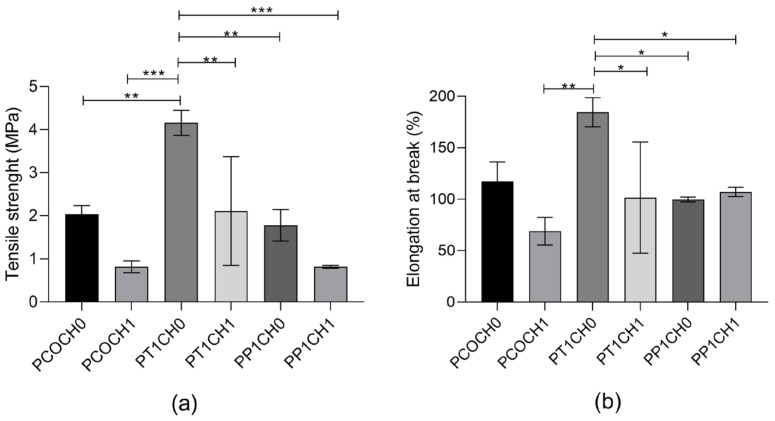
(**a**) Tensile strength and (**b**) elongation at break of PUs. The data are expressed as the mean ± SD (*n* = 3). Rows with * indicates significant differences (*p* < 0.05); ** indicates significant differences (*p* < 0.01); *** indicates significant differences (*p* < 0.001).

The tensile strength of the PUs was in the range of 0.81 MPa to 4.15 MPa (Figure 4a), and elongation at break was between 68.95% to 184.38 (Figure 4b). Differences with the modification of the castor oil can be seen in the tensile strength. The tensile strength was reduced with the incorporation of chitosan into the matrix. This similar behavior is also seen in the elongation at break, with significant differences in the values for the PUs without chitosan and with 1% of chitosan.

**Figure 5:**
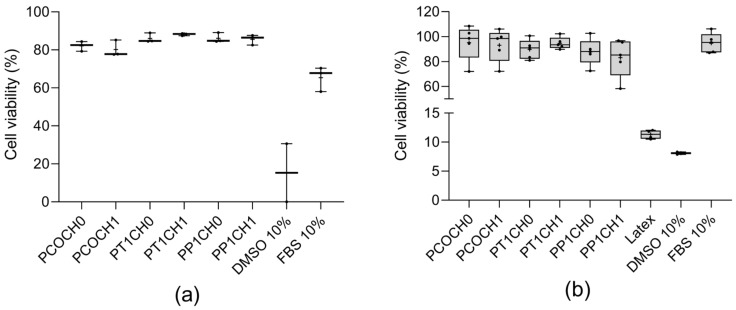
(**a**) L929 viability (%) at 24 h (mean ± SD (*n* = 3)) and (**b**) HDFa viability (%) at 24 h (mean ± SD (*n* = 5)). DMSO as negative control and fetal bovine serum as positive control; + refer to the mean.

For the in vitro evaluation, none of the synthesized PUs were cytotoxic against L929 mouse fibroblasts with cell viabilities up to 80% (Figure 5a) and against human dermal fibroblasts (HDFa) with cell viabilities greater than 80% (Figure 5b). No significant differences were found between the modifications of the polyol or the incorporation of chitosan regarding the cell viability.

**Figure 6:**
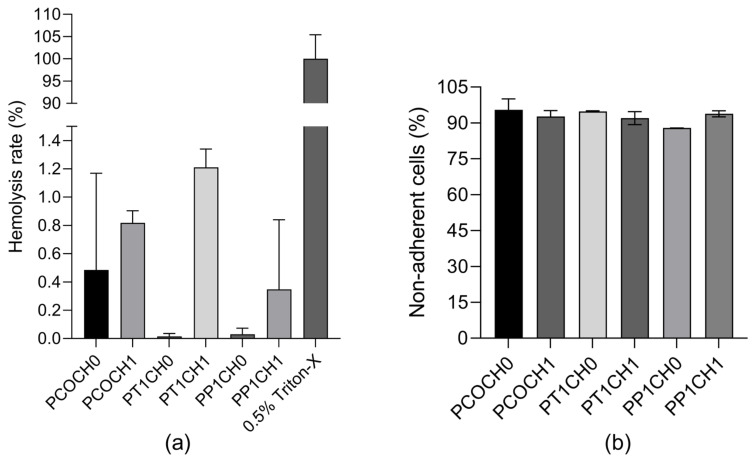
(**a**) Hemolysis rate (%) of PUs (mean ± SD (*n* = 2) and (**b**) non-adherent cells (%) of PUs (mean ± SD (*n* = 2)).

To observe the hemocompatibility of PUs, two tests were performed: hemolysis rate and platelet adhesion. The hemolysis rate was found to be less than 1.21% for all the Pus, and no significant differences were found between the polyurethanes (Figure 6a). And more than 87% of the platelets did not adhere to the PUs (Figure 6b).

## 4. Discussion

Vascular grafts based on synthetic polymers are an interesting solution to autologous ones. However, it has been shown that these can be prone to failure because of a lack of hemocompatibility, and thus, important immune responses, thrombus formation, and neointimal hyperplasia may occur [3]. Due to this, this work shows the synthesis of polyurethanes from castor oil and its modification with TEA and PE to study the effect of the modifications on the mechanical properties, biodegradation, and biocompatibility of the synthesized materials, together with the evaluation of the hemocompatibility of PUs.

As is known, the transesterification of CO modifies the functionality of the polyol by adding functionality with the addition of four hydroxyl groups with PE and three with TEA. This modification is evident in the increase in the hydroxyl index value and in the changes in the water absorption and mechanical properties.

The results show that the modification with chitosan presents a minimum increase in hydrophilicity (Figure 2a) by the addition of polar functional groups (single-bond OH) on its surfaces [16], which may favor the protection of the PU against bacterial adhesion. Although the transesterification of castor oil increases the OH groups, the results do not show a significant change in the hydrophilic character of the PUs with the polyol’s modification. For example, Ahmadi et al. prepared PU scaffolds, and the surface hydrophilicity increased by blending chitosan (2 wt%) with the PU solutions and three different weight ratios of PU/Cs, namely 3:1, 1:1, and 1:3, owing to its hydrophilic nature and positive surface charge [25].

The evaluation of the degradation of PUs is important in the cardiovascular application and the in vivo performance because it is known that a fast degradation implies the loss of the function of the graft, as the mechanical support for the regeneration of the tissue is lost, failing to maintain the shape and the function of the tissue and leading to a failure after implantation [3]. The percentage of enzymatic degradability of PUs does not exceed 0.5%, which is related to the results obtained in the contact angle and water absorption rate tests. Due to the hydrophobic characteristics of PUs synthetized with castor oil and modified castor oil, the interaction between PU and enzymatic media is low, and thus, the mass did not change considerably, indicating a stability of the PUs with the enzymatic media.

Ahmadi et al. evaluated the degradability with PBS over 60 days: the weight of PU and PU/CNT scaffolds was maintained without a measurable loss of weight, but PU scaffolds containing Ch presented a noticeable loss of weight related to their water uptake. As PU and chitosan are hydrophobic and hydrophilic polymers, respectively, the scaffolds containing more chitosan would uptake more PBS [25].

In terms of mechanical properties, the incorporation of chitosan did not modify the mechanical performance of PUs made with unmodified castor oil (Figure 4). The highest values of tensile strength were found for the modification with TEA, and significative differences between the inclusion of chitosan were found for these materials. Both the tensile strength and elongation at break of the PUs increased almost twice with TEA transesterification, while it remained the same with PE transesterification. The transesterification of castor oil made significant differences in the mechanical properties of the PU that can be related to the difference in the hydroxyl value and thus the functionality of the polyol used. Additionally, there was a reduction in the tensile strength with the incorporation of chitosan into the PU with castor oil (form 2.04 ± 0.20 MPa to 0.81 ± 0.14 MPa) and with PE transesterification (from 1.78 ± 0.36 MPa to 0.82 ± 0.03 MPa).

According to this, the inclusion of chitosan in the PU matrix can be seen as a filler that affects the mechanical properties without compromising the biological properties. Biocompatibility is not affected, as it is a surface property, while the mechanical properties are bulk properties. Choi et al. synthesized nanocomposite polymers with waterborne polyurethane (WPU) and chitosan, and for the mechanical properties, they observed that the inclusion of chitosan increased their strength and elongation, and the authors attributed this to the hard and soft segments due to the addition of Ch as a chain extender because the Ch chains are stiffer and can affect the elasticity of the resultant films by cross-linking and hydrogen bonding with CWPU chains [26]. The inclusion of Ch, which is a stiffer molecule, as a filler in the matrix affects the mobility of the chain, which reduces the tensile strength and elongation [7]. Nevertheless, the PUs prepared here pose tensile strengths comparable to the range of human coronary arteries (between 1.40–11.14 MPa) [27].

The thermal degradation of PU occurs in two steps. The first step (around 350 °C) can be related to the degradation of hard segments and decomposition of the urethane linkages; and the second-step degradation, which starts at around 400 °C, could involve the decomposition of ester groups in the polyurethane backbone [14]. All materials show stability above 300 °C, making them suitable for the application as biomaterials, and the PUs are stable in the normal body temperature of 37 °C.

Regarding the cell viability, there was no significant change (*p* < 0.05) in L929 and HDFa viability in PUs obtained from castor oil both unmodified and modified by transesterification. Likewise, the addition of Ch in the polyol did not represent a significant change in cell viability. According to ISO 10993-5, PUs are nontoxic to the L929 and HDFa cells, as the reduction in viability is less than 30%. A sign of cytotoxicity of biomaterials is a reduction in viability of 70% [28].

For L929, the results are in accordance with the literature, where the synthesized PUs are not cytotoxic to the mouse fibroblast. Bahrami et al. prepared PU films using low-pressure plasma treatment and chitosan (1 mg/mL) and studied the biocompatibility of the films by the cell viability of L929, finding that modified films with chitosan showed better cell viability than unmodified films. The highest level of cellular survival (97%) was observed for chitosan-stabilized PU films compared to the control sample [3]. Likewise, Ecevit et al. carried out the functionalization of PU stents with chitosan–fatty acid (CS-FA), evaluated the cytotoxicity of leachables from the prepared materials against L929 murine fibroblast, and found a negligible cytotoxicity of the coating formulations [29].

Similarly, evaluating the cell viability of human dermal fibroblasts on polyurethanes, González-Torres et al., in 2023, prepared chitosan-grafted poly(N-hydroxyethyl acrylamide) (CS-g-PHEAA) and made polyurethanes scaffolds with chitosan and with (CS-g-PHEAA) to support human dermal fibroblasts (HDFs). The PUs were prepared from polyethylene glycol and 1,6-hexamethylene diisocyanate, and the results proved that PUs scaffolds with chitosan are not cytotoxic, and HDFs grown on CS-PU and (CS-g-PHEAA)-PU adopted a rounded shape [30]. Likewise, Porto et al. generated polyurethanes from castor oil as the polyol source, incorporated microcrystalline cellulose (MCC) and cellulose nanocrystals (CNC), and evaluated the cytotoxicity of two PU film extracts obtained after 24 and 168 h of incubation in media with human dermal fibroblast (HDFn) cells; the viability was above 90% for all the materials after 24 h incubation, and the authors reported that the films and composites were noncytotoxic [29]. These results combined with the results present in this paper show the high biocompatibility of castor-oil-based polyurethanes.

For the characterization of biomaterials proposed for blood-contacting applications, hemocompatibility evaluation is indispensable. The hemocompatibility of the PUs was determined by hemolysis rate and platelet adhesion assays. According to the hemolysis rate present, all PUs are in the range of the safe limits for a blood-contacting biomaterial, which is less than 5% according to the ISO10993-4 [20], and according to the ASTM International standards F 756-00, it could be classified as a non-hemolytic material [31].

Amjed et al. prepared chitosan- and starch-based thermoplastic polyurethanes, and the materials showed no significant hemoglobin release; however, the PU with greater chitosan content (20%) presented a higher hemolysis rate (9.5% ± 0.52%) than those without chitosan but with a high content of starch (4.75% ± 0.21%), and all of them presented a high hemolysis rate compared to the PUs synthetized in this study [32].

Similarly, Javaid et al., in 2019, prepared PU with different diisocyanates and hydroxyl-terminated polybutadiene (HTPB) and the inclusion of chitosan. All the chitosan-based PU samples showed greater hemocompatibility as compared to chitosan-free PU samples, and the results suggested that PUs made with chitosan were more compatible with blood cells [33]. Those PU synthesized with IPDI presented 14.71% and 6.01% hemolysis, which is greater than the hemolysis rate obtained in this investigation of PUs from castor oil, modified castor oil, and IPDI.

There was no significance (*p* < 0.05) difference in the platelet adhesions between all the synthesized PU. In vascular grafts, platelet adhesion can be a primary indicator for the formation of thrombosis and inflammation [20]. Thus, biomaterials that do not promote platelet adhesion are desired, as thrombosis is one of the major failure factors in the materials currently used for blood vessel replacement. PUs present less than 15% platelet adhesion, and this can be related to the hydrophobic properties of the surfaces that can cause significant platelet adhesion, which can lead to thrombosis [16]. Given the hydrophilic properties of the PUs synthesized from castor oil and modified castor oil, the addition of chitosan increased the hemocompatibility [16].

The low number of adherent platelets suggests the adequate interaction of castor oil PU with the blood due to the close relationship between thrombus formation and the activation of the coagulation system. As is known, platelet adhesion leads to their activation, which in turn generates the activation of numerous signaling pathways involved in the body’s inflammatory response. Platelet activation implies the release of molecules that will generate further platelet aggregation and thus the formation of the primary thrombus and, as such, the activation of the coagulation system [34]. Therefore, it is necessary to evaluate these factors in the future research of these materials [15,16].

As a limitation of the study, biocompatibility assays were not performed on endothelial cells but were performed on L929 cells obtained from the ATCC, which is a parent L that was derived from normal subcutaneous areolar and adipose tissue of a 100-day-old male C3H/An mouse and is commonly used for the biocompatibility evaluation of new materials [35]. Biocompatibility was also evaluated in HDFa cells, which are a skin cell line with research application in responding to pathogens, skin aging, wound healing, gene delivery, and skin diseases including scleroderma [36]. Also, fibroblast are involved in key processes like breaking down the fibrin clot, creating new extracellular matrix (ECM) and collagen structures to support the other cells associated with effective wound healing and the regeneration of tissue, as well as contracting the wound [37]. Likewise, it is a first indication of the hemocompatibility of the materials when lacking in experiments for the potential activation or non-activation of inflammatory blood cells.

Thus, it was seen that the transesterification of castor oil with PE and TEA generated changes in the properties of the polyurethanes. The modification with TEA affected the hydroxyl index, the water absorption of the polyurethanes, and the mechanical properties. The increase in these properties is favorable for the design of vascular grafts since it allows approaching the values of the native tissue, which could avoid further complications in their use. The castor oil polyurethanes by themselves showed high biocompatibility and hemocompatibility, as they were non-hemolytic, and therefore, the inclusion of chitosan as a filler was not relevant for these properties. However, it did present a decrease in the mechanical properties.

## 5. Conclusions

In terms of the use of these PU in contact with the human body, the aim is to avoid the formation of thrombosis and inflammation and to prevent bacteria that may be in the medium from adhering to the material. The results show that the modification with chitosan presents an increase in the hydrophilicity of the PU, which favors the protection of the material from bacterial adhesion. Although the transesterification of the material increases the hydroxyl groups present in the synthesized material, the results do not show a significant change in the hydrophobic character of the material with the polyol modification. The transesterification of the polyol helps to modify the mechanical properties, making them more suitable for cardiovascular applications without modifying biological properties such as biocompatibility and hemocompatibility. Polyurethanes synthesized with castor oil, oil modifications, and the inclusion of 1% chitosan, when in contact with cells, do not favor cytotoxicity, hemolysis, or platelet adhesion, which makes these materials excellent candidates for use in cardiovascular devices.

## Data Availability

The data presented in this study are available on request from the corresponding author.
